# Application of Spatio-Temporal Context and Convolution Neural Network (CNN) in Grooming Behavior of *Bactrocera minax* (Diptera: Trypetidae) Detection and Statistics

**DOI:** 10.3390/insects11090565

**Published:** 2020-08-24

**Authors:** Zhiliang Zhang, Wei Zhan, Zhangzhang He, Yafeng Zou

**Affiliations:** 1 School of Computer Science, Yangtze University, Jingzhou 434023, China; 201972328@yangtzeu.edu.cn (Z.Z.); 201972326@yangtzeu.edu.cn (Y.Z.); 2 Insect Ecology Laboratory, College of Agriculture, Yangtze University, Jingzhou 434025, China; 201873042@yangtzeu.edu.cn

**Keywords:** *Bactrocera minax*, grooming, image processing, spatio-temporal context, Convolution Neural Network, behavioral sequence

## Abstract

**Simple Summary:**

Traditional manual insect grooming behavior statistical methods are time-consuming, labor-intensive, and error-prone. In response to this problem, we proposed a method for detecting the grooming behavior of *Bactrocera minax* based on computer vision and artificial intelligence. Using this method to detect the grooming behavior of *Bactrocera minax* can save a lot of manpower, the detection accuracy is above 95%, and the difference was less than 15% when compared with the results of manual observation. The experimental results show that the method in this paper greatly reduces the time of manual observation and at the same time ensures the accuracy of insect behavior detection and analysis, which proposes a new informatization analysis method for the behavior statistics of *Bactrocera minax*. At the same time, it also has a positive effect on pest control research.

**Abstract:**

Statistical analysis and research on insect grooming behavior can find more effective methods for pest control. Traditional manual insect grooming behavior statistical methods are time-consuming, labor-intensive, and error-prone. Based on computer vision technology, this paper uses spatio-temporal context to extract video features, uses self-built Convolution Neural Network (CNN) to train the detection model, and proposes a simple and effective *Bactrocera minax* grooming behavior detection method, which automatically detects the grooming behaviors of the flies and analysis results by a computer program. Applying the method training detection model proposed in this paper, the videos of 22 adult flies with a total of 1320 min of grooming behavior were detected and analyzed, and the total detection accuracy was over 95%, the standard error of the accuracy of the behavior detection of each adult flies was less than 3%, and the difference was less than 15% when compared with the results of manual observation. The experimental results show that the method in this paper greatly reduces the time of manual observation and at the same time ensures the accuracy of insect behavior detection and analysis, which proposes a new informatization analysis method for the behavior statistics of *Bactrocera minax* and also provides a new idea for related insect behavior identification research.

## 1. Introduction

Crops and stores have historically been (and will continue to be) attacked by pests [1]. In-depth research on insect behavior can help people learn more about insects, thereby helping to formulate safe and effective prevention strategies. *Bactrocera minax* (Diptera: Trypetidae) is an important citrus pest mainly distributed in China, and it is also one of the targets of external quarantine [2,3,4].

Grooming is a broad definition that covers all forms of body surface care. Grooming is a common and habitual behavior of many insects [5] and is also a very common behavior [5]. Although the insect groups involved in grooming behavior are different, the main functions of grooming behavior are surprisingly similar [6,7]. Remove foreign dust particles from the surface of the epidermis and sensory organs [8], remove body surface secretions and epidermal lipids [9,10], collect pollen particles as food [11], and remove external parasites or pathogens [12]. At the same time, grooming behavior plays a significant role in maintaining the sensitivity of sensory organs [13,14]. As grooming behavior of insects is a very important part of their defense mechanism, it is important to identify and classify these behaviors to help systematically explore the physiological, neurological and pharmacological basis of grooming [6,12,15]. A better understanding of grooming will provide new insight toward the development of control practices, leading to less damage to beneficial insects and consequently new possibilities for sustainable agricultural activity [6].

With the rapid development of computer vision technology, it has become an inevitable trend to use computers to process and analyze video data in various industries to reduce manual labor [16,17]. Computer vision technology has been widely used in our daily life and achieved excellent results, such as face recognition [18] and object detection [19]. These technologies can not only achieve the accuracy of human vision without rest, but are also tens of times faster than manual recognition. The same application also occurs in agriculture. In recent years, agriculture has played a key role in the global economy [20]. The application of computer vision technology in all aspects of agricultural production has higher efficiency than that of manual work, providing a reliable and accurate basis for the regulation and control of agricultural production [20,21]. In the field of agricultural insect behavior analysis, it is the most basic content to establish the behavior spectrum by observing and recording insect behavior [22,23,24]. However, we know that most researchers are still using manual observation and statistical methods to find and record the start time and end time of each behavior by playing the video frame by frame [25]. In this way, it is not only inefficient to find the behavior interval and judge the type manually but also the statistical error problem caused by the increase of personnel fatigue under the condition of long-term observation will gradually increase.

In the course of the experiment, we tried some deep learning algorithms to classify grooming which was developed to track the key parts of the object or predict the behavior [26,27,28], but they are not very suitable in our experimental environment. For example, to identify the grooming behavior of *Bactrocera minax* by tracking the key parts, it is necessary to ensure that the forelegs are visible in most of the time [14]. However, the movement speed of the forelegs in the grooming behavior is very fast, and the mouth grooming and hind leg grooming are often obscured when the fly’s back is facing the camera, similar situations always occur [27]. We hope that after a detection, we can get a complete behavior interval partition result for our subsequent analysis [29], not just real-time detection feedback. 

In this paper, we propose an improved method based on spatio-temporal context and Convolution Neural Network to detect the grooming behavior of *Bactrocera minax*. The background color of *Bactrocera minax* was extracted and the color channel was added into the spatio-temporal feature image. The spatial information of the spatio-temporal feature image was increased, and the distinction between the front grooming (head, foreleg) and the posterior grooming (hind leg, abdomen) of the feature image was enhanced, so the detection model based on CNN can judge the behavior of these images, and achieve the purpose of automatic detection of the grooming behavior of *Bactrocera minax*, and provide a reliable method and idea for improving our ability to document grooming.

## 2. Materials and Methods 

### 2.1. Materials

#### 2.1.1. Development Environment

**Figure 1:**
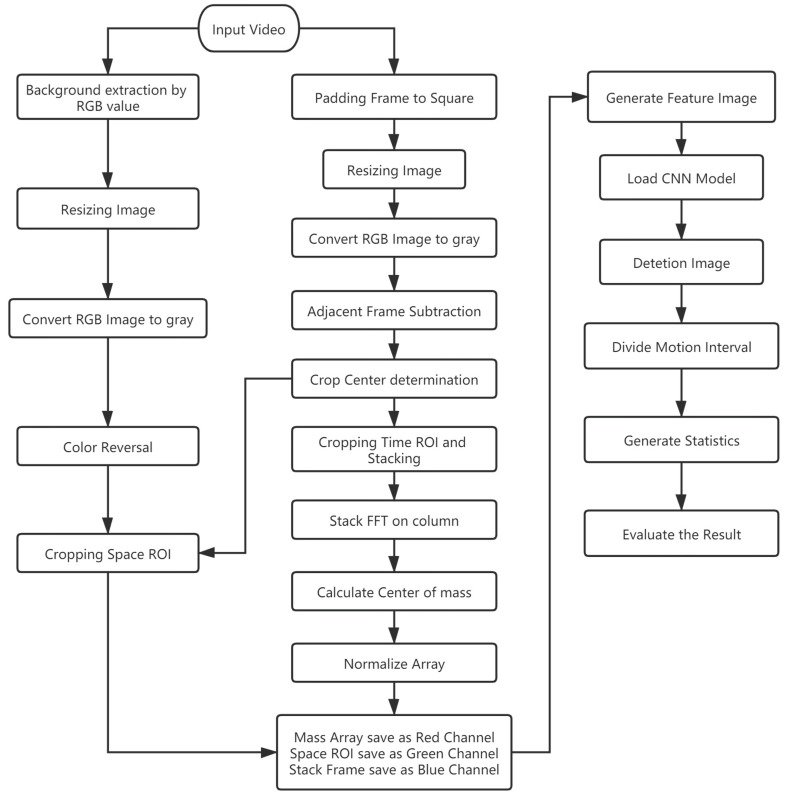
Software flowchart of the overall methodology.

The overall goal of this study was to develop and test a method to detect the grooming behavior of *Bactrocera minax*. The hardware used includes Device 1: Intel core I7 9700 desktop, 16 GB RAM, NVIDIA Geforce RTX 2070 GPU, and Device 2: Intel core I9 9900K desktop, 32 GB RAM, NVIDIA Geforce RTX 2080Ti GPU. Software: Based on Python 3.7.5. Numpy 1.18.0 and OpenCV 4.1.2.30 were used to process videos. Matplotlib 3.1.2 and Pillow 7.0.0 were used to analyze data, and Keras 2.2.5 which based on Tensorflow 1.14.0 was used to train our detection model. Figure 1 shows the software flowchart of the overall methodology used in this paper. We provide the Python code on the GitHub repository (https://github.com/z8446605/GroomingDetection), which can run directly after simple configuration. 

#### 2.1.2. Video Acquisition of *Bactrocera minax*

**Figure 2:**
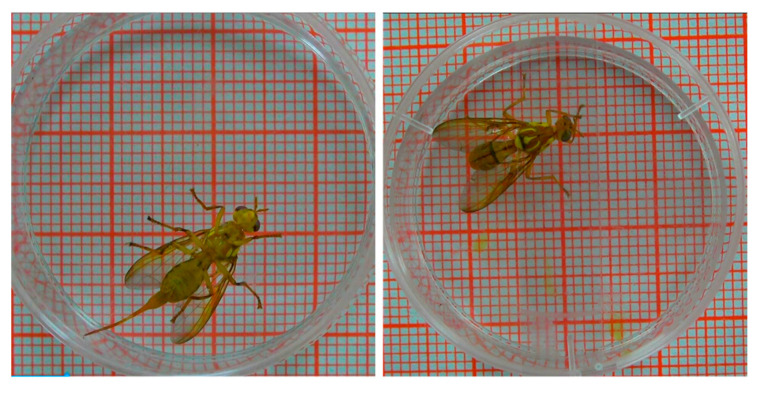
The original video frames of *Bactrocera minax*. The original video was cut, only the middle petri dish was reserved. Size: (35 × 20 mm), the *Bactrocera minax* adults were allowed inactivity, walking and grooming.

Acquisition date: May–August 2019, Videos were taken by Sony HXR-MC58C digital camera, video resolution: 1920 × 1080, frame rate 25 fps, the recording time of each *Bactrocera minax* adult was 60 min. The original video effect is shown in Figure 2. 

### 2.2. Videos Processing

#### 2.2.1. Color Extraction of *Bactrocera minax*

According to the color components of the background, the red (R), green (G), blue (B) threshold is determined between low = [90, 50, 30] and high = [255, 255, 255]. According to the threshold, the background is extracted (Equation (1)), and the extracted part is transformed into gray which contains the information of background, we call it *P_b_* (Equation (2)). Then reverse the image color, the new image we called image *S* (space) containing only the pixel information of the insect body is obtained (Equation (3)).

(1) $$P_{b}^{xy}=\{\begin{array}{l} 0,\;\left[R,\;G,B\right]<low\;or\;\left[R,G,B\right]>high\\ 255,\;low<\left[R,G,B\right]<high\; \end{array},$$

(2) $$P_{b}=\left[\begin{array}{ccc} p_{b}^{11} & \cdots & p_{b}^{1y}\\ \vdots & \ddots & \vdots \\ p_{b}^{x1} & \cdots & p_{b}^{xy} \end{array}\right],$$

(3) $$S=\left[\begin{array}{ccc} 255 & \cdots & 255\\ \vdots & \ddots & \vdots \\ 255 & \cdots & 255 \end{array}\right]-P_{b},$$

**Figure 3:**
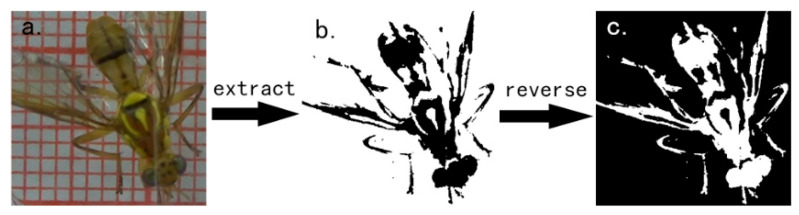
The extraction process of body part information of *Bactrocera minax*. (**a**) The original video frame contains the *Bactrocera minax* and the background which is RGB image; (**b**) Image *P_b_* only contains background, the gray value of the black region is 0, and the gray value of the white region is 255; (**c**) Image *P_b_* after reversal.

Mark *x* is the row index, mark *y* is the column index, *x* = 0, 1, 2..., 1919, *y* = 0, 1, 2…, 1079, the extraction process is shown in Figure 3.

#### 2.2.2. Spatial Information Extracting and Frame Cropping

Converting RGB image to gray and padding 0 pixels to make it square, then resizing the image to 500 × 500. By detecting the gray level change of each pixel in the time window (*w*) of 7 frames (0.28 s), the possible behavior in the video will be found [27], the gray value of frame *F*_*i*−1_ has subtracted from frame *F_i_* and accumulated to calculate the gray change value in *i* frames (Equation (4)).

(4) $$D=\sum_{i=1}^{w}ABS\left(F_{i}-F_{i-1}\right),$$

In Equation (4), the result *D* is a matrix which size is 500 × 500, *i* = 1, 2, 3…, *w* − 1, the value of *i* is the frame index, *w* is the time window range of which is 1 to 7. Then we use the function *argmax* () in Numpy to return the coordinate {*x_c_*, *y_c_*} of the point with the maximum gray change (Equation (5)).

(5) $$\left\{x_{c},\;y_{c}\right\}=argmax\left(D\right),$$

Taking this coordinate as the center to crop a 100 × 100 ROI in each time window [27], we call it Time ROI (T-ROI). At the same time, the image *S* in Equation (3) is also cropped with the coordinate as the center, so that the spatial information and the temporal information can be consistent, we call this Space ROI (S-ROI). 

#### 2.2.3. Temporal Features Extracting

Converting 7 frames in *T-ROI* to row vectors (Equation (6)), matrix *T* has 7 rows and 10,000 columns, subscript *w* is the time window, subscript *p* represents the index number of the pixels, *p* = 1, 2, 3…, 10,000.

(6) $$T=\left[\begin{array}{cc} \begin{array}{cc} t_{11} & t_{12}\\ t_{21} & t_{22} \end{array} & \begin{array}{cc} \cdots & t_{1p}\\ \cdots & t_{2p} \end{array}\\ \begin{array}{cc} \vdots & \vdots \\ t_{w1} & t_{w2} \end{array} & \begin{array}{cc} \vdots & \vdots \\ \cdots & t_{wp} \end{array} \end{array}\right]$$

Then we process *T* on columns by fast Fourier transformation (FFT) to obtain Fourier transform matrix *F_t_* (Equation (7)), *ABS* stands for absolute value, *f_ti_* is a row vector. With this method, we can mark the position of the pixel whose gray value changes greatly in these 7 frames, the smaller the change of gray value, the closer the transformation value is to 0.

(7) $$F_{t}=ABS\left(FFT\left(T\right)\right)=ABS\left[\begin{array}{c} ft_{1}\\ \begin{array}{c} ft_{2}\\ \begin{array}{c} \vdots \\ ft_{w} \end{array} \end{array} \end{array}\right]$$

Calculate the center of mass (*m_p_*) of each pixel on columns for *F_t_* (Equation (8)), *i* = 1, 2, 3…, 7, *p* = 1, 2, 3…, 10,000.

(8) $$m_{p}=\frac{\sum_{i}ift_{ip}}{ft_{ip}}$$

Finally, the vector *M* containing the center of mass *m_p_* of each pixel *p* is obtained (Equation (9)).

(9) $$M=\left[\begin{array}{cc} \begin{array}{cc} m_{1} & m_{2} \end{array} & \begin{array}{cc} \cdots & m_{p} \end{array} \end{array}\right]$$

#### 2.2.4. The Combination of Spatial Information and Temporal Features

The vector *M* is reconstructed into the center of a mass matrix of ROI size, and normalize the elements in matrix *M*, the element values are limited between 0 and 1 (Equation (10), which use the function *clip* () in Numpy.

(10) $$M_{N}=clip\left(M,\;0,\;1\right)$$

Finally, save *M_N_* as a red channel, select the second index frame of *T* in the matrix as a blue channel, and save *S-ROI* as the green channel (Equation (11)).

(11) $$\{\begin{array}{l} red=255M_{N}\\ green=SROI/1.8\\ blue=255t_{2} \end{array}$$

### 2.3. Generate the Detection Model

**Figure 4:**
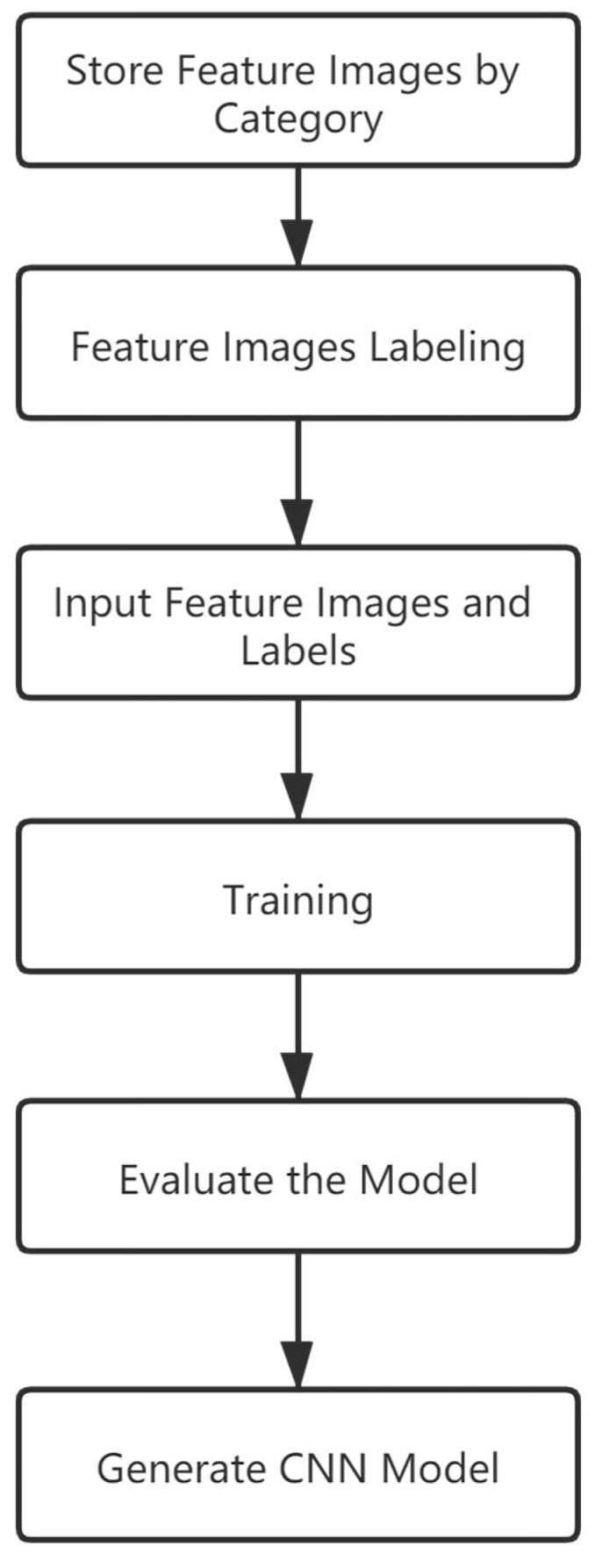
The process of labeling and training.

We create a training set and put it into the CNN for training, Figure 4 shows the process of labeling and training. 

#### 2.3.1. Selecting Feature Images to Create a Training Set

The labeled videos contain 20 *Bactrocera minax* adults with a total of 1200 min, the video shooting time of each *Bactrocera minax* was 60 min. First, we use the method described in Section 2.2 to process these videos to generate their feature images. According to the characteristics of the generated feature images, the behavioral feature images are divided into seven types: Foreleg, head, hind leg, abdomen, wing, mid and motionless, head grooming includes feeler grooming, mouth grooming, and eye grooming, mid grooming includes all the front and posterior grooming behaviors participated by the middle legs. Then we classified the behavior of these feature images by human vision, and the feature images of different grooming behavior stored in the corresponding folder, which can effectively collect a large number of effective behavior feature images.

The number of feature images for each grooming behavior is shown in Table 1, with a total of 30,508 images. 

Then, the classified feature images are renamed by behavior name and number (such as head_1, head_2 ... head_5202), to facilitate management in the future. Setting the label number corresponding to each behavior in advance, and we use the Regular Expression Operations (re) in Python to match the image name to determine the behavior label number of the images, and save the images path and its labels in a .csv file.

#### 2.3.2. Training Model

**Figure 5:**
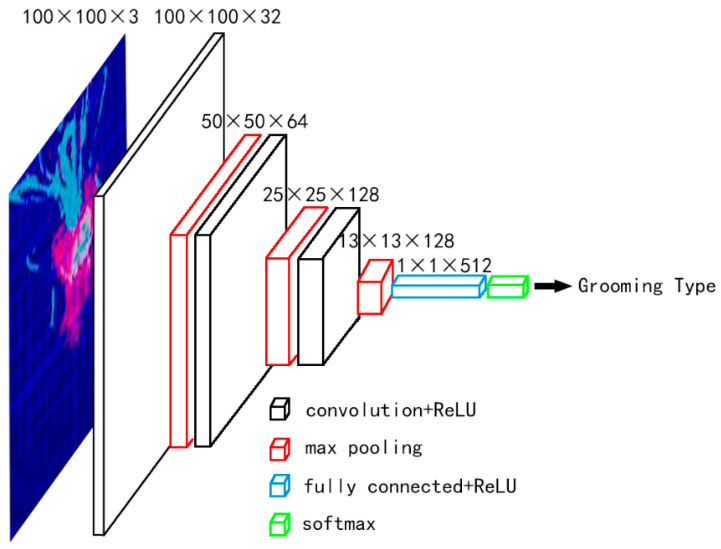
The CNN structure adopted in this paper.

The CNN is used to train, which structure is shown in Figure 5. 

The network contains 3 convolution layers, 3 max-pooling layers, 1 fully connected layer, and 1 softmax output. The convolution kernel size is 3 × 3 because the 3 × 3 convolution is the smallest size that can capture the eight domain information of a pixel. The limited receptive field of two 3 × 3 stacked roll bases is 5 × 5, and that of three 3 × 3 stacked roll bases is 7 × 7, thus it is possible to replace the large-scale one by stacking the small-scale one, the size of the receptive field remained unchanged. The advantage is that 3 × 3 convolution has fewer parameters than other convolutions with large size [28].

After each convolution, one max-pooling is performed. The pooling kernel size is 2 × 2, the stride is 2. So after each pooling, the output image size will be reduced by half (round up). One fully connected after 3 times pooling. Finally, use softmax to output the behavior classification. 

We divided 30,508 feature images into a training set and validation set at a ratio of 7 to 3. The initial learning rate is 0.01, we used Adam optimizer, the loss function is categorical_crossentropy, the batch size is 256, and trained the network 10 epochs to obtain the detection model. 

### 2.4. Analyzing the Detection Result and Generating Statistics

Firstly, the method in Section 2.2 is used to process the video to be analyzed and generate its corresponding feature image. Then use the detection model trained in Section 2.3 to detect each feature image, and the detection model will output the behavior detection result of the feature image corresponding to each frame. Finally, the frame index and the behavior detection results corresponding to the frame are stored in the list for the next analysis. According to the detection results of each frame, the corresponding interval of each action will be divided. Because the grooming behavior of *Bactrocera minax* adults lasts at least 0.5 s, so we set that only the behavior that lasts at least 10 frames (0.4 s) will be counted. If the same grooming behavior is not detected in the next 10 frames, the behavior is considered to have ended, and from the first frame of these 10 frames, the current behavior is judged again. Occasionally, on the one hand, the generated feature image will be distorted in several frames, which will lead to failure of behavior detection. On the other hand, the detection results of the convolution network model will be wrong. Combined with our extensive observations, we set this judgment threshold to 10 frames, which not only ensures that grooming behaviors with short duration will not be missed but also ensures that behaviors will not be recorded once more in case of accidental detection errors. Once the behavior change is detected (for 10 consecutive frames), the starting time point of the behavior should return to 10 frames before to ensure that the starting time statistics are correct (10 frames have occurred in this behavior). During the detection process, the start time, end time, and behavior type of each behavior will be recorded in real-time.

## 3. Results

### 3.1. Grooming Behavior of *Bactrocera minax* Adults

Grooming behaviors include leg grooming, wing grooming, feeler grooming, eye grooming, mouth grooming, and abdomen grooming. The *Bactrocera minax* adults grooming the mouth, feelers and eyes with two forelegs, grooming wings, and abdomen with two hind legs. The two forelegs rub each other, and the two hind legs act the same to complete the grooming. The two middle legs are fixed and they cannot rub with each other, and only one middle leg can rub with forelegs or hind legs [25]. 

**Figure 6:**
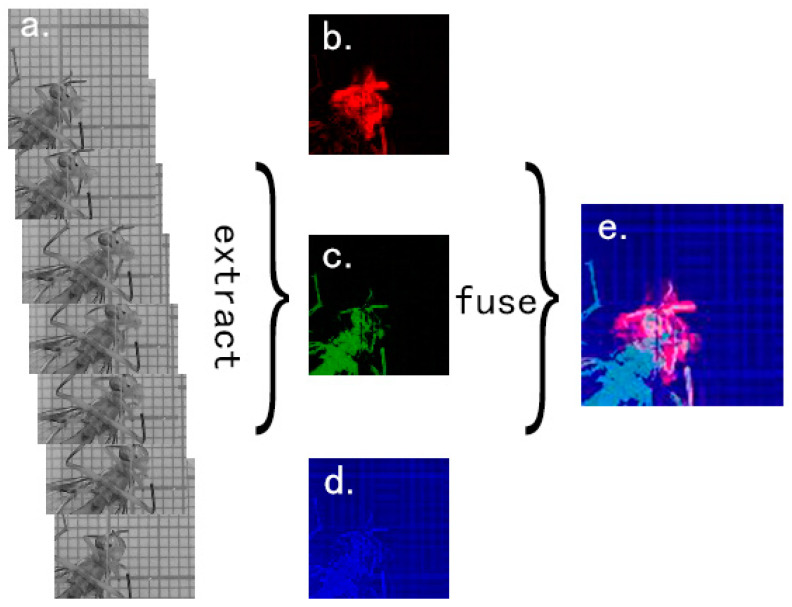
Examples of feature image generation. (**a**) The 7 original gray images based on 7 original RGB frames; (**b**) Temporal features (T-ROI) of *Bactrocera minax* generated by 7 frames time window based on Figure 6a; (**c**) Spatial information (S-ROI) of *Bactrocera minax* extracted by the second image in Figure 6a; (**d**) Static information generated after normalization of the gray value of the second image in Figure 6a; (**e**) The final feature image which contains temporal features, spatial information, and static information.

In this paper, the original RGB frames of *Bactrocera minax* was processed in two steps, the spatial information and temporal features of *Bactrocera minax* were fused into a new feature image, using CNN detection model to classify the grooming type. Figure 6a shows the original gray images. Figure 6b–d show temporal features, spatial information, and static information, respectively. Figure 6e shows the final feature image.

**Figure 7:**
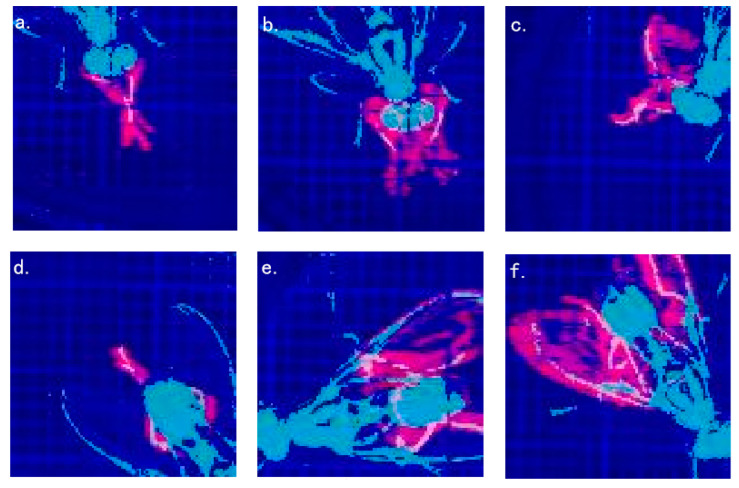
The result of each behavior feature image; (**a**) Foreleg grooming; (**b**) head grooming; (**c**) foreleg grooming with the participation of middle leg; (**d**) hind leg grooming; (**e**) abdomen grooming; (**f**) wing grooming.

As shown in Figure 7, the feature images generated by each behavior have obvious differences. After training in the convolutional neural network, each kind of behavior can be effectively classified. 

### 3.2. Detection of the Grooming Behavior

**Figure 8:**
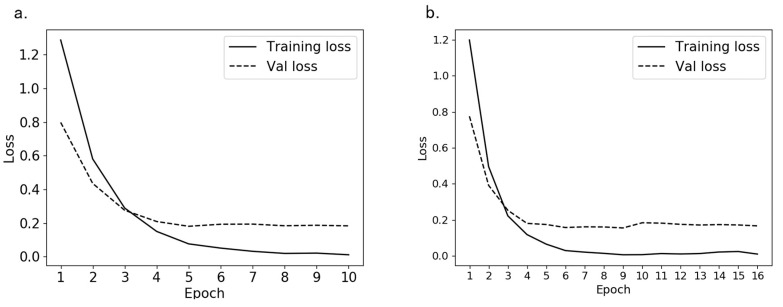
The loss reduction during the training process; (**a**) training 10 epochs loss reduction; (**b**) training 16 epochs loss reduction.

After labeling 30,508 feature images of *Bactrocera minax*, we trained 10 and 16 epochs according to the method we proposed in Section 2.3.2, the loss reduction during the training process is shown in Figure 8. It can be seen that the loss of training set and validation set in the first five epochs decreased rapidly, and at 6 or 7 epochs, the best effect was almost achieved. And it can be seen from Figure 8b that even if the epochs of training is increased, the loss value tends to stabilize, indicating that the CNN model has converged. 

**Figure 9:**
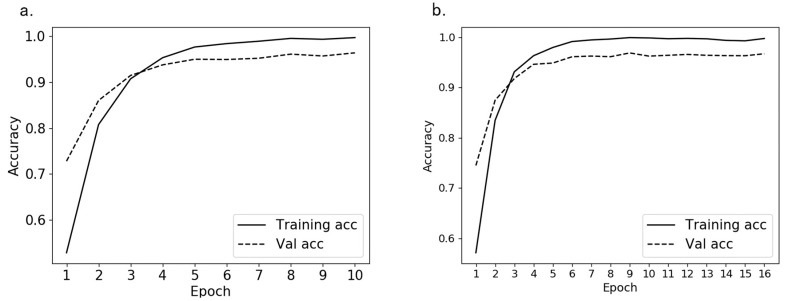
The accuracy of the training set and validation set in the training process; (**a**) the accuracy of training 10 epochs change; (**b**) the accuracy of training 16 epochs change.

The accuracy during the training process is shown in Figure 9. We can get the rise of accuracy corresponding to the loss reduction. From the comparison of Figure 9a,b, it can be seen that the accuracy of the validation set is finally stable at about 96.5%, which shows that the model training is completed under the parameters we set.

**Figure 10:**
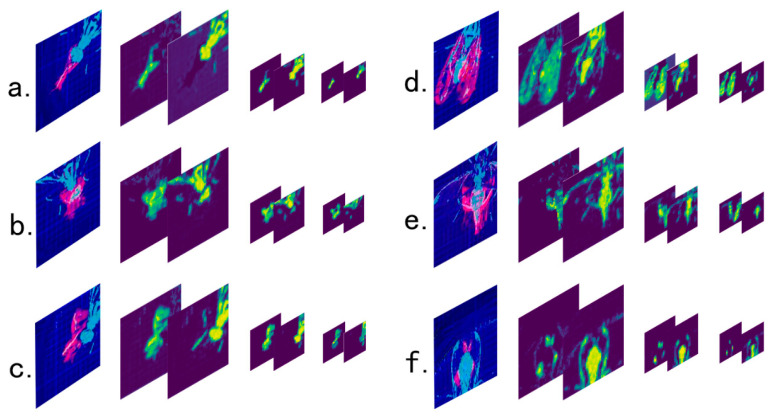
Feature map of the six grooming behaviors. Figure (**a**–**f**) are the spatio-temporal feature images of foreleg grooming, head grooming, mid grooming, wing grooming, abdomen grooming and hind leg grooming, and neural network feature maps extracted by our trained detection model. The first image of each group is the original feature image, the second is the temporal feature map obtained after the first pooling of neural network, the third is the spatial feature map obtained after the first pooling, the fourth is the temporal feature map obtained after the second pooling, the fifth is the spatial feature map obtained after the second pooling, the sixth is the temporal feature map obtained after the third pooling, and the seventh is the spatial feature map obtained after the third pooling.

After training, the detection model can extract the different features of every grooming behavior. Figure 10 shows the CNN feature map of the six grooming behaviors. Figure 10a–c shows the front grooming, with similar spatial information, but obvious differences in temporal features. Figure 10d–f shows the posterior grooming, it also has similar spatial information and different temporal features. The complete feature map of the CNN model after the third pooling corresponding to each grooming behavior is shown in Figures S1–S6.

A total of 22 adults of *Bactrocera minax* which were not in the training set were selected, and the video shooting time of each *Bactrocera minax* was 60 min, which was divided into five videos. The statistical accuracy of most flies is above 95%, and the standard error of statistical accuracy among individuals is 2.88%, which shows that the system is robust. The specific results are shown in Table 2 and Table 3. We tested and verified the accuracy by observing the behavior detection interval with human eyes, that is, the judgment of grooming behavior interval will be regarded as correct only if it reaches the standard of manual recognition. Table S1 shows the detection results of each video.

### 3.3. Statistical Differences

**Figure 11:**
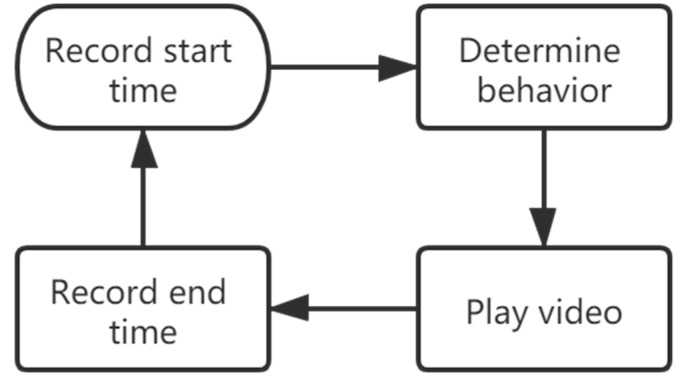
The process of manual record of one grooming behavior.

The process of a manual record of one grooming behavior is shown in Figure 11. The duration of one grooming behavior of *Bactrocera minax* adults is 0.5–60 s. It is undoubtedly time-consuming to manually search for the start and end time of grooming behavior. If the duration of grooming behavior is short, it may need to repeatedly watch and confirm, and if the duration of grooming behavior is long, it becomes more difficult to find the end time. 

**Figure 12:**
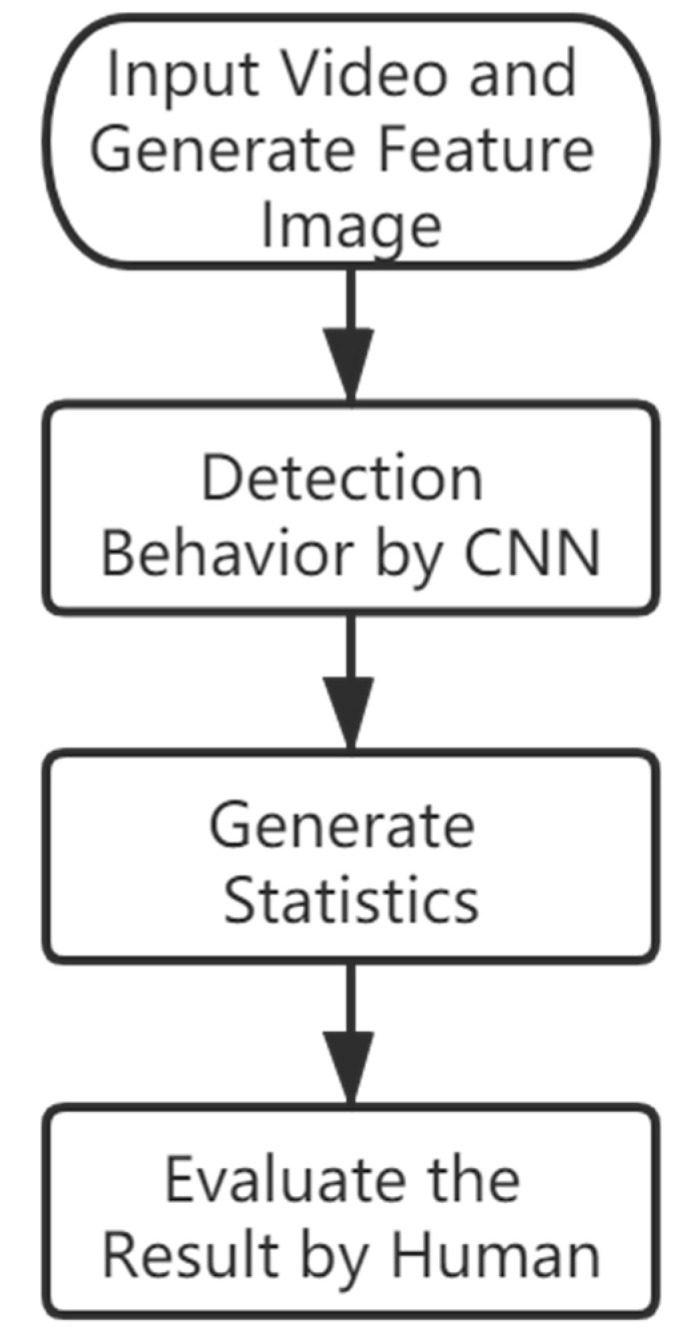
Recording process of the grooming behavior in this paper.

The method introduced in this paper can make up for this defect. The specific process is shown in Figure 12. In addition to manual verification, the other three steps are all automatically run by the set program and get the results. In the process of manual verification, it is very easy and efficient to judge the behavior, and the manual recording of grooming behavior takes several times of the system.

We selected 10 videos that are not in the training set and used both manual statistics and our program at the same time, and each video lasted 12 min. Then, we compare the difference between software statistics and manual statistics to verify the availability of our proposed method. Different results, such as behavior types, the difference of behavior duration interval is more than 15 frames, and different numbers of behaviors are counted in the same period (for example, in two seconds, our program thinks that there are two behaviors, but only one behavior is counted manually). These differences will be recorded as one difference (the numerator in Equation (12)). Finally, we calculated the difference between the two methods (Equation (12)).

(12) $$Difference=\frac{Different\;number\;of\;results}{Total\;number\;of\;behaviors\;counted\;manually}$$

The difference was between 10% and 15%, and the specific difference results are shown in Table 4.

### 3.4. Comparison of Several Detection Methods

We compared the accuracy and convergence speed of validation sets of four different detection methods (Table 5). The training set and validation set used by the four detection methods are the same, the optimizer used in training is Adam, the loss function is categorical_crossentropy, the initial learning rate is 0.01, the batch size is 256, and 16 epochs are trained. The results show that the recognition rate is improved and the convergence speed is faster when the proposed method is used to detect the grooming behavior of *Bactrocera minax*. Optimization comes from two aspects. First, we optimized the generation process of feature images, which made the feature recognition of the grooming behavior of *Bactrocera minax* higher. The second is the optimization design of neural network structure. Compared with vgg16, our network has much fewer parameters, which ensures accuracy and gives consideration to the performance.

The accuracy of each epoch of we proposed method is shown in Figure 9b, and the accuracy of each epoch of the other three detection methods are shown in the supplementary materials (Figure S7).

### 3.5. Performance

We tested the performance of the program on two experimental devices and detected 10 videos using the complete process in this paper. Each video was 12 min and 30 s. The average feature image generation rate and feature image detection time are shown in Table 6. The generated feature image is completely calculated by the CPU, while the detection feature image is mainly calculated by the GPU. 

## 4. Discussion

The rapid development of agricultural insect recognition and animal pose estimation based on computer vision has inspired us to develop a reliable statistical system for the grooming behavior of *Bactrocera minax*. We’re going to process video data more efficiently. At present, computer vision technology has been widely used in agricultural research [30,31,32,33], such as crop pest detection [34,35,36] or pest activity detection [37], crop disease detection [38], identification of crop growth [39,40], crop yield prediction [41], and animal behavior detection [26,27,42]. The first four kinds of applications can get good results by processing and analyzing only a few clear images. Such as Ulzii-Orshikh Dorj et al.’s method for predicting fruit yield in 2017 [41], by transforming and processing the original RGB image, the fruit target was separated from the background image. A similar approach is used in our paper, when there is a difference between the target and the background color information, this traditional and simple method is effective. Animal behavior detecting is the analysis of video streams, and the size of the target, the state and the recording environment have a greater impact on the detection effect. So we need to optimize the detection method for *Bactrocera minax*, the final experimental results also verify the effect of our experiment.

Identification of fruit fly adult species based on machine vision [43] or the method of identifying and counting other insects [44,45,46] is mature and has practical applications. At present, using the popular deep learning object detection algorithm, as yolo [47,48] and maskRCNN [49,50] identification can also achieve better results. There are a few methods to detect the grooming behavior of flies. One is to analyze video using deep neural networks, such as DeepLabCut and LEAP. The former uses Deep Residual Networks (ResNet), a small number of labeled images were put into training to predict the key parts of the body [51], and the latter trained hundreds of markers to predict the location of the target body, classification of behaviors through unsupervised learning [26]. These two methods based on the deep neural network have been used in Drosophila experiments, with good results, and have strong generalization. However, the low overall quality of video and the small proportion of the objects in the video and the rapid movement of the observation site limit our use of the method. Our experiments are different from the two, the *Bactrocera minax* is relatively small in the video, and grooming will occur motion blur or mutual occlusion between parts, resulting in key points difficult to predict. This is mainly due to the lack of information on the spatial scale of video frames. Although the above two methods have achieved better results using deeper network structures, for our experimental environment, the loss of pooling information and the gradient disappearance problem in the deep neural network [52], simply deepening the network depth cannot continue to improve the accuracy [53].

Benefit from the study of the spatio-temporal context in predicting human or animal behavior, we can use temporal information to complement insufficient spatial information. Inspired by ABRS [27], we can create better behavioral spatio-temporal features of *Bactrocera minax*. Through the fusion of spatial information and temporal features, a more intuitive spatio-temporal feature image is generated, so that the behavior category to which the feature image belongs can be directly judged by the human eye or computer vision. We choose CNN for feature image detection, not only because of its reliability in the field of computer vision [54,55], but also because of the convenience brought by the large collection of methods based on current CNN library, labeling, training, and prediction are very easy to understand for people who are not in this field, and the results are clear.

Finally, we can achieve more than 95% accuracy after a small amount of manual verification, and the difference between the results of complete manual statistics is stable between 10%~15%, which indicates that the final result is credible and stable. Part of the difference is due to differences in judgment, such as three specific behaviors in head grooming, which are somewhat ambiguous in multiple observations. The other part is that the system is more sensitive to the boundaries of behavior, and complete manual observation may not be sensitive enough to the front grooming behavior of less than 1 s, which is common in the experimental process. This behavior generally involves head grooming and foreleg grooming. After a long time of work, completely manual work may not be able to find out this difference every time, but machines can. 

Our next research direction is to further optimize the way of generating spatio-temporal feature images. On the one hand, we consider optimizing the use of CPU multi-threading when the program is running, or using GPU to accelerate the processing of video frames and subsequent FFT to achieve a faster detection speed than the current one [56,57]. The improvement in performance means that we can retain more video frame details during the frame cropping process, thereby improving the quality of the spatio-temporal feature image generation. This also means using deeper or larger CNN structure, such as the current high robust ResNet model provides the possibility [53]. On the other hand, we may use the Fully Convolutional Network (FCNN) [58] or U-Net [59] to segment the object from the background to achieve better results than RGB features segmentation. The former proposes an end-to-end fully convolution network for semantic segmentation, which combines deep and coarse network layer semantic information with shallow and fine network layer surface information to generate accurate segmentation [58]. And the latter improves FCNN, it has a large number of feature channels in the up-sampling part, which allows the network to propagate context information to higher resolution layers [59]. In short, the further optimization of each step of the method provides a basis for the method to be used in a wider range in the future.

## 5. Conclusions

We optimize the detection method for the grooming behavior of *Bactrocera minax* adults, which uses the background and target extraction method to separate the object, and fuses them with the features based on temporal context extraction. The CNN model is used to detect the fused feature images, and a reliable statistical method is designed for grooming behavior law of *Bactrocera minax* adults. The final detection effect is good, the average detection accuracy is above 95%, and the standard error is below 3%. The statistical efficiency is greatly improved compared with the complete manual statistical method. The detection results are ideal and the detection effect is stable, and the difference between the statistical results and the manual results is within the controllable range, which accords with the error requirement of the research experiment on the grooming behavior of the *Bactrocera minax* adults. In the detection of the grooming behavior of the *Bactrocera minax*, compared with other methods, the performance of the model is guaranteed with higher accuracy. Several specific methods are proposed for the next step of the study, which provide a theoretical basis for further improving the performance and detection accuracy of the program.

## Figures and Tables

**Table 1:** The number of feature images for each grooming behavior.

Behavior Type	Counts	Behavior Type	Counts
Foreleg grooming	5464	Head grooming	5202
Mid grooming	4502	Abdomen grooming	3702
Hind leg grooming	3486	Wing grooming	4140
Motionless	4012		

**Table 2:** Average accuracy and standard error.

Total Number of Behaviors	Average Accuracy	Standard Error
7893	95.71%	2.88%

**Table 3:** Accuracy of behavior detection results.

*Bactrocera minax* Number	Number of Behaviors	Accuracy	Deviance	*Bactrocera minax* Number	Number of Behaviors	Accuracy	Deviance
1	379	97.89%	2.18%	12	417	91.37%	−4.34%
2	324	99.69%	3.98%	13	237	99.16%	3.45%
3	378	93.12%	−2.59%	14	406	90.89%	−4.82%
4	336	96.13%	0.42%	15	218	97.71%	2.00%
5	584	96.58%	0.87%	16	558	93.73%	−1.98%
6	492	95.33%	−0.38%	17	53	96.23%	0.52%
7	186	93.01%	−2.70%	18	397	94.71%	−1.00%
8	550	97.27%	1.56%	19	328	98.78%	3.07%
9	300	98.33%	2.62%	20	121	96.69%	0.98%
10	594	96.63%	0.92%	21	247	97.17%	1.46%
11	329	97.26%	1.55%	22	459	88.02%	−7.69%

**Table 4:** Statistical differences.

Video Number	Total Number of Behaviors Counted Manually	Different Number of Results	Difference Degree
1	134	20	14.93%
2	140	15	10.71%
3	50	8	16.00%
4	66	6	9.09%
5	36	5	13.89%
6	94	12	12.77%
7	115	13	11.30%
8	88	7	7.95%
9	79	12	15.19%
10	102	11	10.78%

**Table 5:** Comparison of several detection methods.

Detection Methods	Validation Set Accuracy	Convergence Speed on RTX 2070 GPU (s/epoch)
Method 1 ^1^	92.23%	3.19
Method 2 ^2^	95.28%	7.13
Method 3 ^3^	95.30%	44.75
Method 4 ^4^	96.68%	7.25

^1^ The complete method proposed by Primoz Ravbar et al. in 2019 (ABRS) [27]; ^2^ Feature images generated by ABRS, detection model trained by the method in Section 2.3.2; ^3^ Feature images generated by the method in Section 2.2, detection model trained by vgg16 [28]; ^4^ The complete method we proposed in this paper.

**Table 6:** The performance of the program.

Device 1: I7 9700 CPU, RTX 2070 GPU, 16GB RAM			Device 2: I9 9900K CPU, RTX 2080Ti GPU, 32GB RAM		
Video Number	Feature Image Generation Rate (fps/s)	Feature Image Detection Time (s)	Video Number	Feature Image Generation Rate (fps/s)	Feature Image Detection Time (s)
1	15.16	18.74	1	16.23	11.12
2	14.88	17.28	2	15.72	10.43
3	14.82	17.99	3	15.69	10.03
4	14.87	19.15	4	15.64	9.63
5	14.91	18.00	5	16.01	8.29
6	14.56	18.22	6	15.52	10.25
7	14.65	18.67	7	15.71	9.81
8	15.01	21.95	8	15.98	9.76
9	14.96	15.89	9	15.92	9.72
10	14.89	15.29	10	15.81	9.92

## Supplementary Materials

The following are available online at https://www.mdpi.com/2075-4450/11/9/565/s1, Figure S1: The complete feature map after the third pooling corresponding to foreleg grooming, Figure S2: The complete feature map after the third pooling corresponding to mid grooming, Figure S3: The complete feature map after the third pooling corresponding to hind leg grooming, Figure S4: The complete feature map after the third pooling corresponding to head grooming, Figure S5: The complete feature map after the third pooling corresponding to abdomen grooming, Figure S6: The complete feature map after the third pooling corresponding to wing grooming, Table S1: The detailed video detection results corresponding to each adult flies, Figure S7: The accuracy of each epoch of the other three detection methods.
