# Supplementary material for: Application of Spatio-Temporal Context and Convolution Neural Network (CNN) in Grooming Behavior of Bactrocera minax (Diptera: Trypetidae) Detection and Statistics
Source: Insects. 2020 Aug 24;11(9):565. doi: 10.3390/insects11090565 (PMC7564701; doi:10.3390/insects11090565)
Supplement: Supplementary file 1 [file insects-11-00565-s001.pdf]

insects

MDPI

Table S1. The detailed video detection results corresponding to each adult flies.

Video number	Number of behaviors	Accuracy	Video number	Number of behaviors	Accuracy
1_1	140	96.43%	12_1	215	88.37%
1_2	144	99.31%	12_2	42	85.71%
1_3	62	100.00%	12_3	59	98.31%
1_4	33	93.94%	12_4	75	96.00%
2_1	58	100.00%	12_5	26	96.15%
2_2	58	100.00%	13_1	65	100.00%
2_3	44	100.00%	13_2	52	100.00%
2_4	92	98.91%	13_3	29	96.56%
2_5	72	100.00%	13_4	27	100.00%
3_1	49	100.00%	13_5	64	98.44%
3_2	76	93.42%	14_1	103	88.35%
3_3	24	91.67%	14_2	92	93.48%
3_4	108	90.74%	14_3	124	87.10%
3_5	121	92.56%	14_4	36	97.22%
4_1	24	100.00%	14_5	51	96.08%
4_2	102	94.12%	15_1	75	96.00%
4_3	69	97.10%	15_2	9	88.89%
4_4	64	96.88%	15_3	103	99.03%
4_5	77	96.10%	15_4	14	100.00%
5_1	121	96.69%	15_5	17	100.00%
5_2	153	98.04%	16_1	227	92.51%
5_3	122	95.90%	16_2	102	92.16%
5_4	142	95.07%	16_3	69	94.20%
5_5	46	97.83%	16_4	72	95.83%
6_1	192	93.75%	16_5	88	96.59%
6_2	167	98.80%	17_1	1	100.00%
6_3	40	87.50%	17_2	33	96.70%
6_4	82	95.12%	17_3	3	100.00%
6_5	11	100.00%	17_4	16	94.12%
7_1	65	95.38%	18_1	53	96.23%
7_2	28	67.86%	18_2	65	89.23%
7_3	51	100.00%	18_3	115	91.30%
7_4	9	100.00%	18_4	123	100.00%
7_5	33	96.97%	18_5	41	95.12%
8_1	84	92.86%	19_1	71	100.00%
8_2	128	96.09%	19_2	1	100.00%
8_3	227	100.00%	19_3	19	100.00%
8_4	15	100.00%	19_4	126	98.41%
8_5	96	95.83%	19_5	111	98.20%
9_1	42	100.00%	20_1	1	100.00%
9_2	92	97.83%	20_2	101	96.04%
9_3	65	98.46%	20_3	8	100.00%
9_4	34	97.06%	20_4	11	100.00%
9_5	67	98.51%	21_1	40	92.68%
10_1	89	93.26%	21_2	86	97.67%
10_2	158	98.10%	21_3	48	100.00%
10_3	76	97.37%	21_4	73	97.26%

10_4	72	98.61%	22_1	101	87.13%
10_5	199	95.97%	22_2	92	90.22%
11_1	101	94.06%	22_3	65	81.54%
11_2	72	95.83%	22_4	152	88.82%
11_3	150	100.00%	22_5	49	91.84%
11_4	6	100.00%			

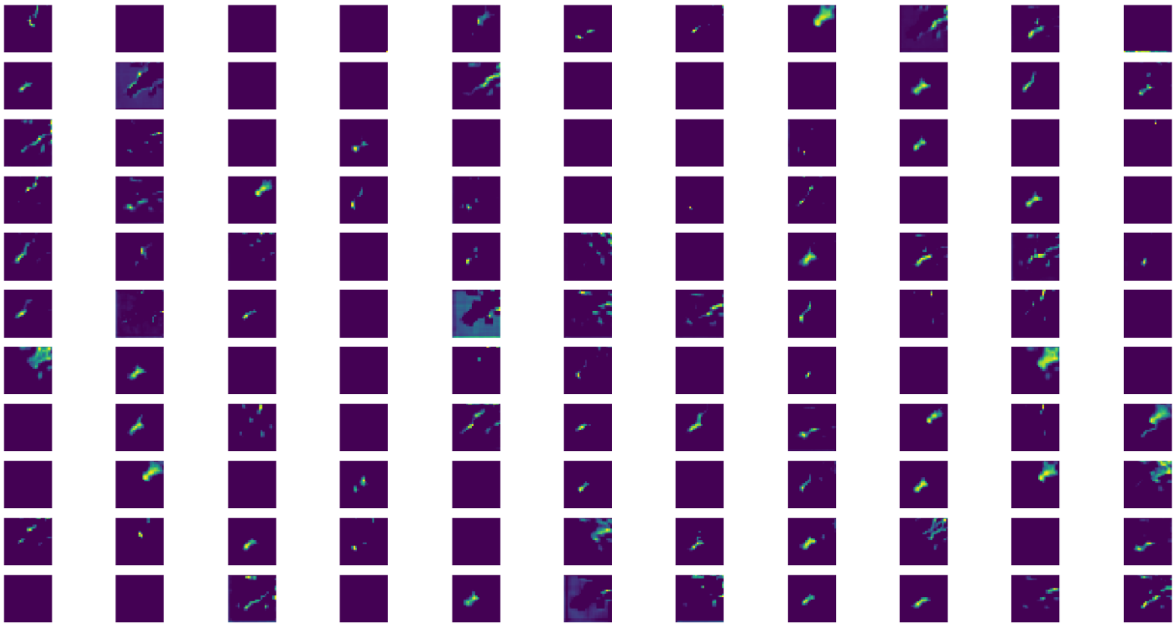

Figure S1. The complete feature map of the CNN model after the third pooling corresponding to foreleg grooming.

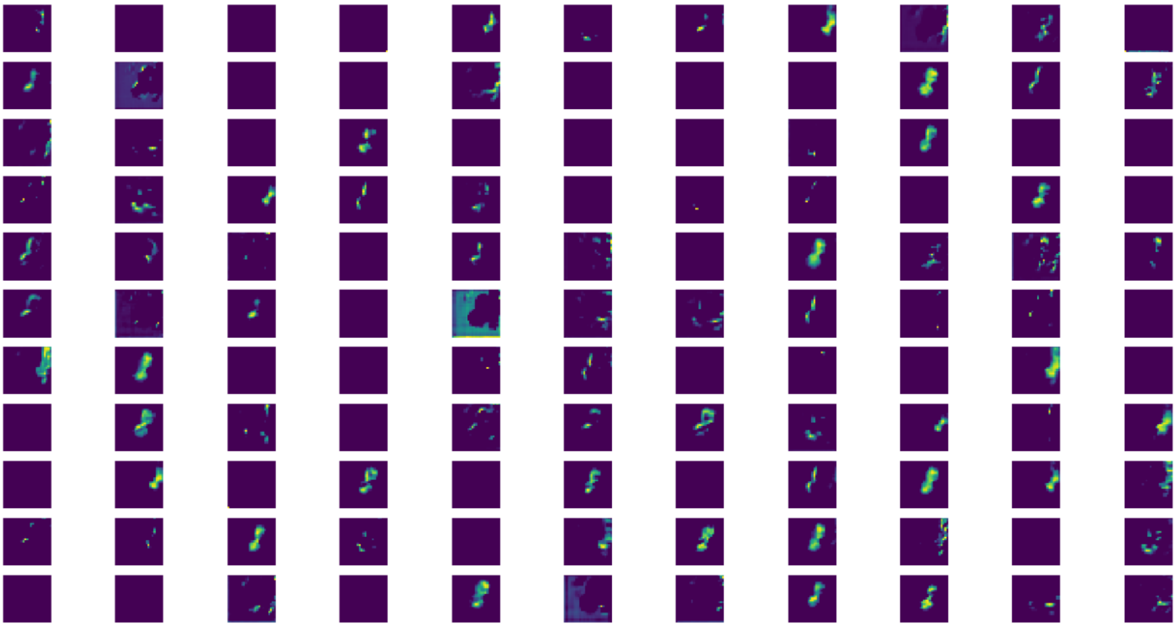

Figure S2. The complete feature map of the CNN model after the third pooling corresponding to mid grooming.

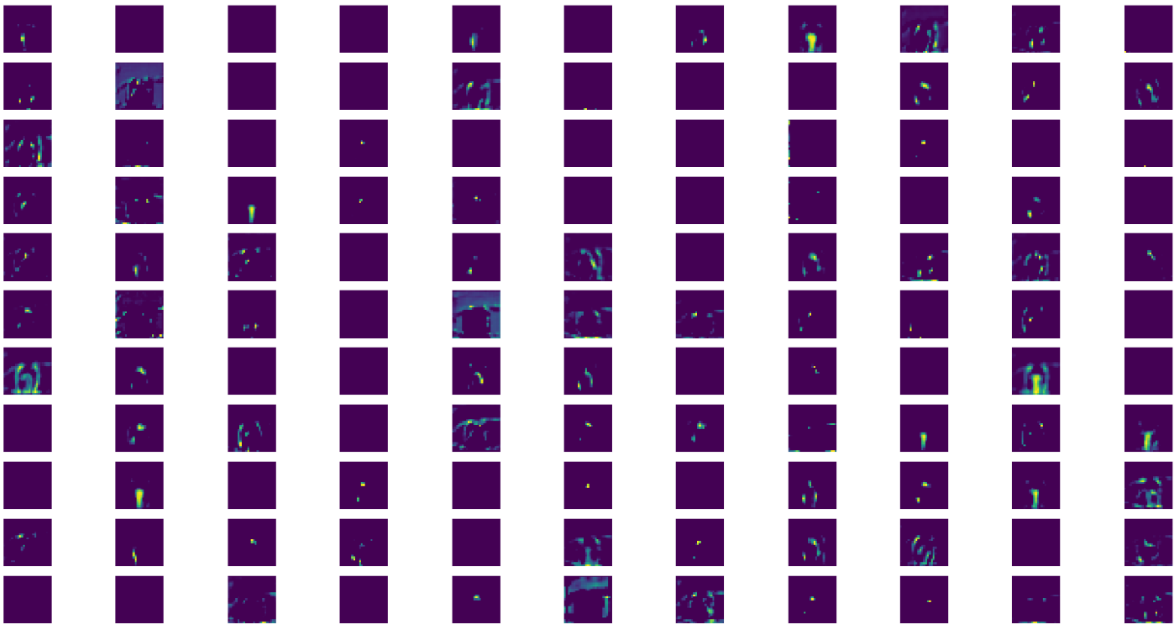

Figure S3. The complete feature map of the CNN model after the third pooling corresponding to hind leg grooming.

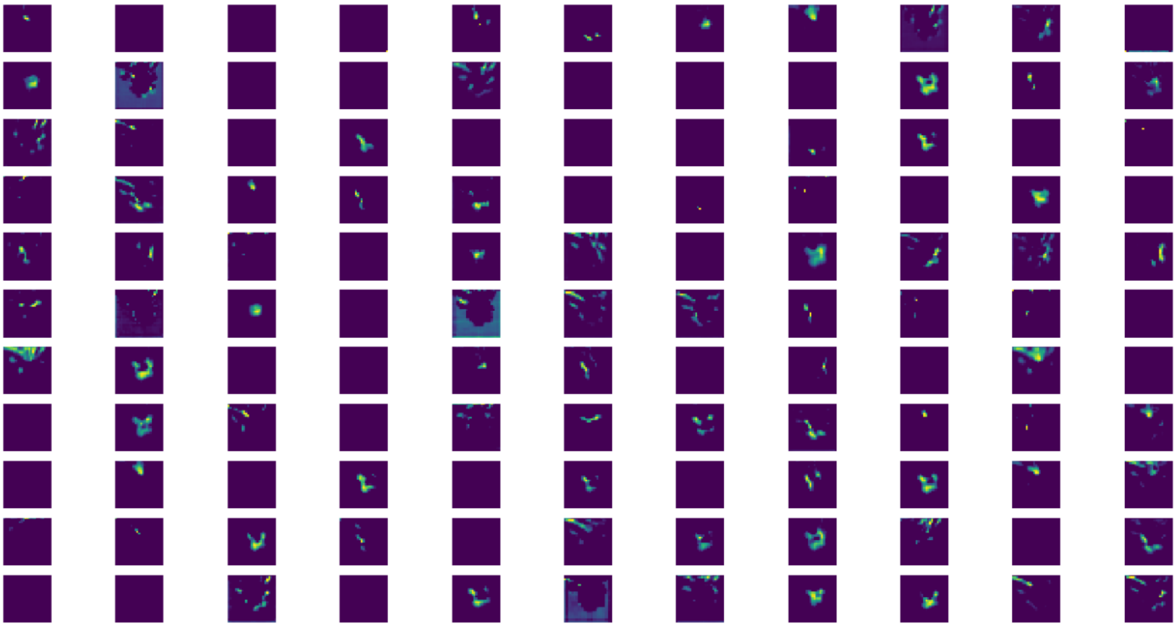

Figure S4. The complete feature map of the CNN model after the third pooling corresponding to head grooming.

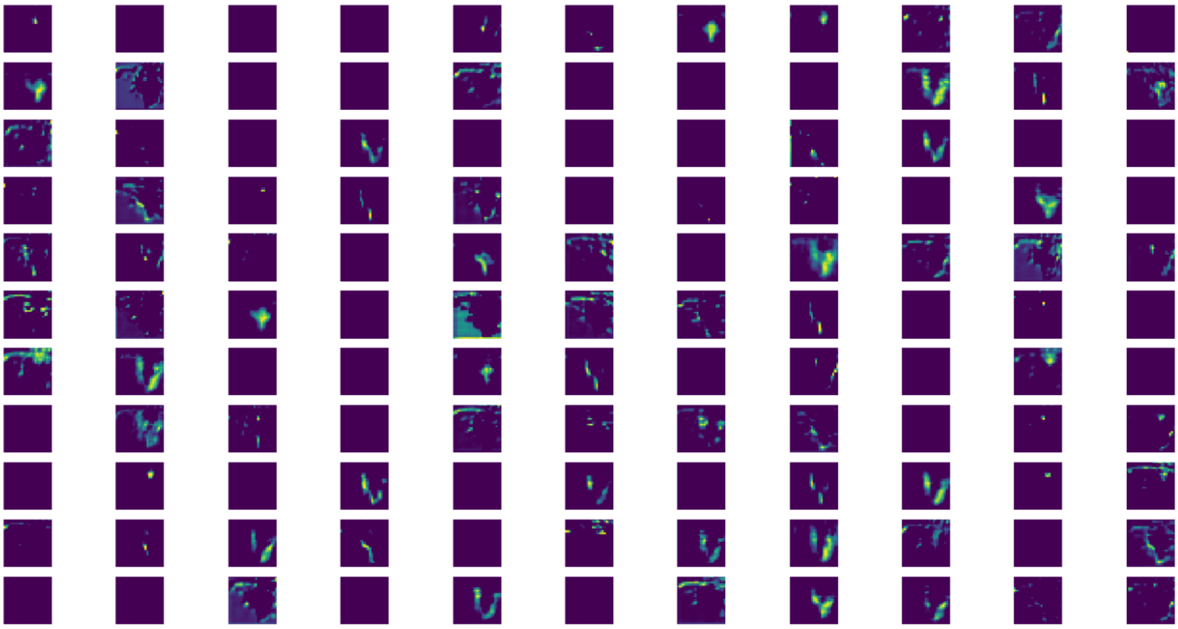

Figure S5. The complete feature map of the CNN model after the third pooling corresponding to abdomen grooming.

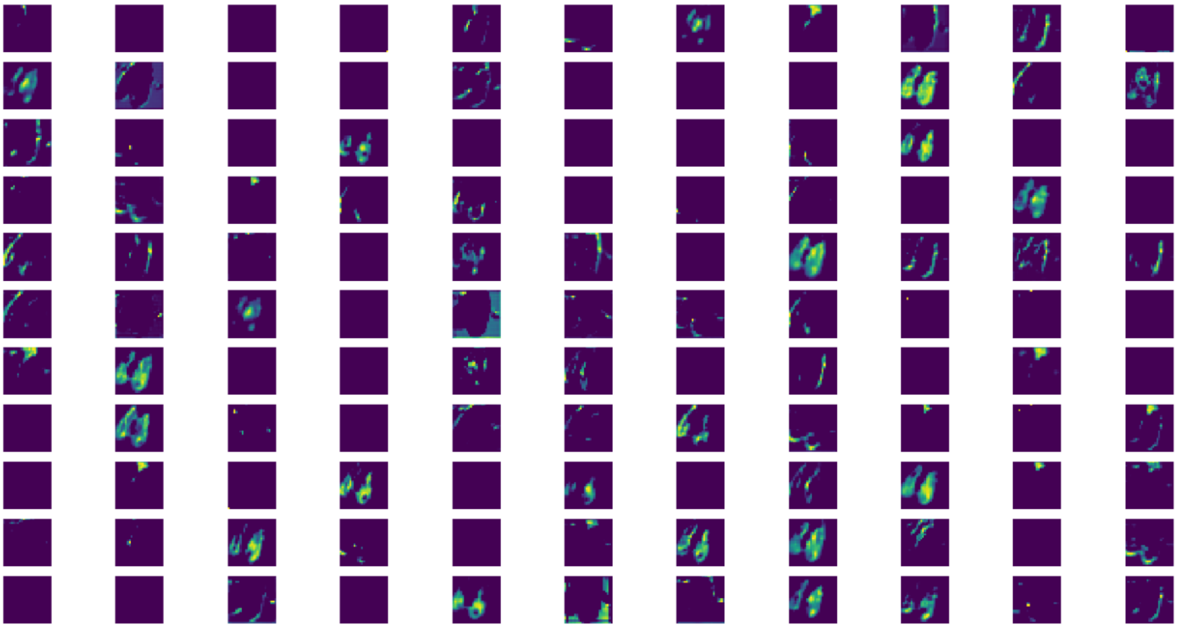

Figure S6. The complete feature map of the CNN model after the third pooling corresponding to wing grooming.

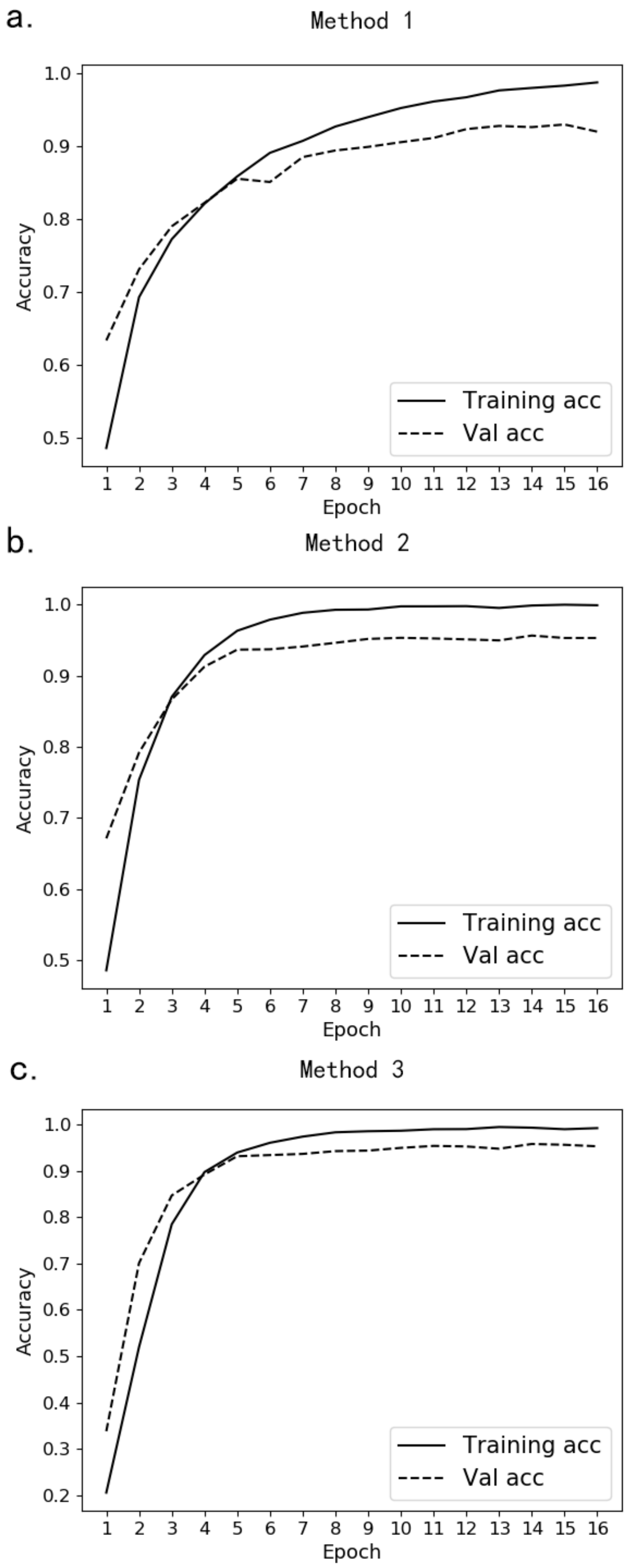

Figure S7. The accuracy of each epoch of the other three detection methods. (**a**) The complete method in ABRS; (**b**) Feature images generated by ABRS, detection model trained by the method in 2.3.2; (**c**) Feature images generated by the method in 2.2, detection model trained by vgg16.
